# Upregulation of lncRNA LANCL1-AS1 inhibits the progression of non-small-cell lung cancer via the miR-3680-3p/GMFG axis

**DOI:** 10.1515/med-2023-0666

**Published:** 2023-03-13

**Authors:** Hui Pan, Jing Peng, Xiaoni Qiao, Han Gao

**Affiliations:** Department of Respiratory Medicine, Gansu Provincial Hospital, Lanzhou 730000, Gansu, China; Department of Customer Service Center, Gansu Provincial Hospital, Lanzhou 730000, Gansu, China; Department of Information Center, Gansu Provincial Hospital, Lanzhou 730000, Gansu, China

**Keywords:** non-small-cell lung cancer, LANCL1-AS1, miR-3680-3p, GMFG

## Abstract

Patients with non-small-cell lung cancer (NSCLC) have a low survival rate. Long non-coding RNA (LncRNA) LANCL1 antisense RNA 1 (LANCL1-AS1) was indicated to be downregulated in NSCLC; however, its detailed function in NSCLC is unanswered. Real-time quantitative polymerase chain reaction revealed the downregulation of LANCL1-AS1 in NSCLC cell lines and subcellular fractionation assay showed that LANCL1-AS1 was mainly located in the cytoplasm of NSCLC cells. Cell counting kit-8, Transwell, and tube formation assays displayed that overexpression of LANCL1-AS1 suppressed NSCLC cell proliferation, migration, invasiveness, and angiogenesis *in vitro*. Animal experiments validated the tumor-suppressive role of LANCL1-AS1 in tumor-bearing mice. Mechanistically, LANCL1-AS1 upregulated glia maturation factor gamma (GMFG) expression by competitively binding to miR-3680-3p. GMFG knockdown reversed LANCL1-AS1 overexpression-mediated inhibitory impact on NSCLC malignant behaviors. Collectively, LANCL1-AS1 upregulation inhibits the progression of NSCLC by modulating the miR-3680-3p/GMFG axis.

## 1 Introduction

Lung cancer (LC) is the second most prevalent malignancy and the major cause of cancer-related deaths [1]. It is estimated in 2022 that LC accounts for 21% of all cancer-related deaths in men and women in the United States [2]. Emerging evidence has indicated that smoking is the main predisposing factor of LC [3]. Small-cell lung cancer (SCLC) and non-small-cell lung cancer (NSCLC) are two major types of LC, and NSCLC mainly incudes lung adenocarcinoma (LUAD) and lung squamous cell carcinoma (LUSC), accounting for more than 80% of all LC cases [4]. Despite the declines in the incidence and mortality of LC owing to therapeutic advances and cessation, the 5-year relative survival rate of LC patients is 21%, while that of patients with metastatic disease is only 6% [5]. Hence, finding a more effective biomarker for the diagnosis and therapy of LC is urgent.

Long non-coding RNAs (lncRNAs) are RNA segments consisting of over 200 nucleotides which have no potential to encode proteins but have critical functions in various cellular processes by interacting with downstream molecules [6]. Plentiful studies have verified the involvement of lncRNAs in the tumorigenesis, pathogenesis, and angiogenesis of cancers, including NSCLC [7,8]. For example, LINC01599 is considered as an oncogene in LUAD by promoting autophagy [1]. LINC01296 knockdown inhibits the progression of NSCLC via the miR-143-3p/ATG2B [9]. Furthermore, the regulatory functions of lncRNAs are determined differently by their subcellular location. Numerous studies demonstrated that in the cytoplasm, lncRNAs work as competing endogenous RNAs (ceRNAs) to regulate downstream RNA expression [10]. LANCL1 antisense RNA 1 (LANCL1-AS1) is a novel lncRNA which was reported to be downregulated in NSCLC [11]. Additionally, LANCL1-AS1 was indicated to be an autophagy-related RNA in LUAD [1]. Bioinformatics analysis elucidated the downregulation of LANCL1-AS1 in lung tumors compared with adjacent normal tissues, indicating that LANCL1-AS1 might be a tumor suppressor of LC. In addition, lncLocator (http://www.csbio.sjtu.edu.cn/bioinf/lncLocator/) predicted that LANCL1-AS1 is largely distributed in the cytoplasm. Nevertheless, the detailed function of LANCL1-AS1 in NSCLC is unanswered.

This study aimed to probe the role as well as the regulatory mechanism of LANCL1-AS1 in NSCLC. We hypothesized that LANCL1-AS1 might act as a ceRNA to affect the pathogenesis of NSCLC by modulating downstream molecule expression. Our findings might help to develop a new idea for the diagnosis and therapy of NSCLC.

## 2 Materials and methods

### 2.1 Cell culture and transfection

Human normal bronchial epithelial cell line (HBE) and NSCLC cell lines (A549, H1299, and H460) were obtained from the Cell Bank of Chinese Academy of Sciences (Shanghai, China) and incubated in RPMI-1640 medium (Gibco, Waltham, MA, USA) containing 10% fetal bovine serum (FBS; Invitrogen, Carlsbad, CA, USA), 100 U/mL penicillin, and 100 mg/mL streptomycin (Gibco) at 37°C with 5% CO_2_ in a humidified incubator. For cell transfection, pcDNA3.1/LANCL1-AS1 and empty pcDNA3.1 vector were synthesized by GenePharma (Shanghai, China) for overexpression assays. For the downregulation of miR-3680-3p and glia maturation factor gamma (GMFG), miR-3680-3p inhibitor or the negative control (NC inhibitor) and short hairpin RNAs targeting GMFG (sh-GMFG#1/2) or sh-NC were also obtained from RiboBio (Guangzhou, China). The above vectors were transfected into A549 and H460 cells, respectively using Lipofectamine 2000 (Invitrogen). Cells transfected for 48 h were used for subsequent analysis.

### 2.2 Real-time quantitative polymerase chain reaction (RT-qPCR)

Total RNA was isolated from NSCLC cells or tumor tissues of mice using TRIzol reagent (Invitrogen). The cDNA was synthesized by reverse transcription of 1 μg RNA samples using SuperScript II (Vazyme, Nanjing, China). RT-qPCR was implemented with SYBR Premix Ex Taq II (Takara, Dalian, China) on a 7500 Real-Time PCR System (Applied Biosystems, Carlsbad, CA, USA). Quantification of LANCL1-AS1, mRNAs, and miRNAs was conducted with the 2^−ΔΔCt^ method, normalized to GAPDH and U6, respectively. Primer sequences are listed in Table 1.

**Table 1:** Primer sequence used for RT-qPCR

Gene	Sequence (5′→3′)
LANCL1-AS1 forward	TGGATCTTCAGCAGCCACCT
LANCL1-AS1 reverse	TCCAGGCCTGATAGTGTAGCT
miR-6748-3p forward	ACACTCCAGCTGGGTCCTGTCCCTGTCTC
miR-6748-3p reverse	TGGTGTCGTGGAGTCG
miR-3680-3p forward	ACACTCCAGCTGGGTTTTGCATGACCCTGGG
miR-3680-3p reverse	TGGTGTCGTGGAGTCG
miR-214-5p forward	ACACTCCAGCTGGGTGCCTGTCTACACTTG
miR-214-5p reverse	TGGTGTCGTGGAGTCG
miR-4732-5p forward	ACACTCCAGCTGGGTGTAGAGCAGGGAGCAG
miR-4732-5p reverse	TGGTGTCGTGGAGTCG
KCNMB2 forward	GCACCGGATCGCTGTCATTA
KCNMB2 reverse	TGGCAAAAAGACCTCCGGTA
TFRC forward	GGTTATGTGGCGTATAGTAAG
TFRC reverse	CTGAGTGTGATTGAAGGAAG
GMFG forward	AAAGAAGAGGCCTGTGGACAG
GMFG reverse	TGGTTGTTCAGGTCCTAGGG
GAPDH forward	TATGATGATATCAAGAGGGTAGT
GAPDH reverse	TGTATCCAAACTCATTGTCATAC
U6 forward	GTGCGTGTCGTGGAGTCG
U6 reverse	AACGCTTCACGAATTTGCGT

### 2.3 Subcellular fractionation assay

Nuclear/Cytosol Fractionation Kit (Biovision, Shenzhen, China) was used for nuclear and cytoplasmic fraction extraction from A549 and H460 cells. LANCL1-AS1 were isolated from the two fractions and quantified with RT-qPCR. GAPDH and U6 were used as endogenous controls for cytoplasm and nucleus, respectively.

### 2.4 Cell counting kit-8 (CCK-8) assay

NSCLC cells were placed into 96-well plates (1 × 10^3^ cells/well). At 24, 48, and 72 h, CCK-8 solution (10 μL, Dojindo, Kumamoto, Japan) was added, and the cells were cultured for further 2 h at 37°C. Afterwards, the absorbance at 450 nm was assessed with a microplate reader (Thermo Scientific, Waltham, MA, USA). For cell sensitivity assessment, NSCLC cells were treated with IC50 of gefitinib (a tyrosine kinase inhibitor, TKI drug; MedChemExpress, Shanghai, China) for 24 h, followed by adding CCK-8 solution.

### 2.5 Transwell assay

Transwell assays were implemented for measuring cell migratory or invasive capabilities. NSCLC cells (2 × 10^4^) were added to the upper chamber of the Transwell chamber (8 μm pore size; Corning, Lowell, MA, USA). The lower or upper chamber was added with complete medium or serum-free medium, respectively. After 48 h of incubation, the non-migratory cells in the upper chamber were swabbed and the migratory cells were subjected to 0.1% crystal violet staining. The invasion assay was similar except that the upper chamber was precoated with Matrigel (BD Biosciences, San Jose, CA, USA). Stained cells were imaged under a microscope (Nikon, Tokyo, Japan).

### 2.6 Tube formation assay

Human umbilical vein endothelial cell line (HUVEC) was obtained from American Type Culture Collection (ATCC, Manassas, VA, USA) and incubated in Endothelial Cell Growth Medium BulletKit (EGM, Lonza, Switzerland) at 37°C with 5% CO_2_ in a humidified atmosphere. HUVECs (2 × 10^4^) in 200 μL conditional medium from A549 and H460 cells were inoculated into a 24-well plate which was precoated with Matrigel (Corning) followed by incubation with 5% CO_2_ at 37°C for 6 h. Tube structures were photographed under a bright-field microscope (Nikon). The mesh and length of the completed tubes were measured to quantify tube formation using Image View 3.7 (Jingtong, China).

### 2.7 Western blotting

Proteins were isolated from cells using RIPA lysis buffer (Thermo Scientific) and quantified with a BCA assay kit (Bio‐Rad, Hercules, CA, USA). Equal amounts of protein samples (20 μg) were dissolved by 10% SDS-PAGE (Bio-Rad) and blotted onto polyvinylidene fluoride (PVDF) membranes (GE Healthcare, Beijing, China). Membranes were blocked with 5% defatted milk and incubated at 4°C overnight with primary antibodies against fibroblast growth factor 2 (FGF2, ab208687), vascular endothelial growth factor (VEGF, ab46154), angiopoietin 1 (Ang1, ab183701), β-actin (ab115777) (all from Abcam, Cambridge, MA, USA) and GMFG (13625-1-AP; Proteintech, Chicago, IL, USA), followed by incubation with the secondary antibody (ab7090, Abcam) at room temperature for 2 h. The blots were visualized with the ECL kit (Vazyme, Nanjing, China) and quantified with ImageJ software (GE Healthcare).

### 2.8 Luciferase reporter assay

Putative binding site between LANCL1-AS1 and miR-3680-3p or miR-3680-3p and GMFG was predicted by DIANA (http://carolina.imis.athena-innovation.gr/diana_tools/web/) and TargetScan (http://www.targetscan.org/vert_71/), respectively. Putative binding site was mutated by Phusion Site-Directed Mutagenesis Kits (Thermo Scientific). Wild type (Wt) or mutant (Mut) miR-3680-3p or GMFG was synthesized and subcloned into pmirGLO vectors (Promega, Madison, WI, USA) to construct miR-3680-3p-Wt/Mut and GMFG-Wt/Mut. Then, miR-3680-Wt/Mut and GMFG-Wt/Mut were co-transfected with pcDNA3.1/LANCL1-AS1 (or empty pcDNA3.1) and miR-3680-3p inhibitor, respectively, into A549 and H460 cells using Lipofectamine 2000 (Invitrogen). Forty-eight hours later, the luciferase activity was measured with a dual luciferase^®^ reporter assay system (Promega).

### 2.9 *In vivo* xenograft experiments

BALB/c nude mice (male, 5–6 weeks) were obtained from Vital River (Beijing, China) and randomly divided into two groups (*n* = 5 per group). Recombinant lentivirus carrying LANCL1-AS1 (Lv-LANCL1-AS1) and empty control vector (Lv-con) were synthesized by GenePharma. Mice were injected subcutaneously with H460 cells (2 × 10^5^) expressing Lv-LANCL1-AS1 or Lv-con. Tumor volume was monitored every 3 days and computed by the formula: volume = ½(length × width^2^). On 18th day, the mice were sacrificed under anesthesia and tumor weight was measured. All animal experiments implemented were approved by the Ethics Committee of Gansu Provincial Hospital.

### 2.10 Statistical analysis

Data are expressed as the mean value ± standard deviation (SD). Statistical analysis was conducted using SPSS 21.0 (IBM Corp., Armonk, NY, USA). Student’s *t*-test was utilized for two group comparison, while analysis of variance (ANOVA) was utilized for multiple group comparison followed by Tukey’s *post hoc* analysis. *p* < 0.05 was regarded to be statistically significant.

## 3 Results

### 3.1 LANCL1-AS1 is downregulated in NSCLC

**Figure 1:**
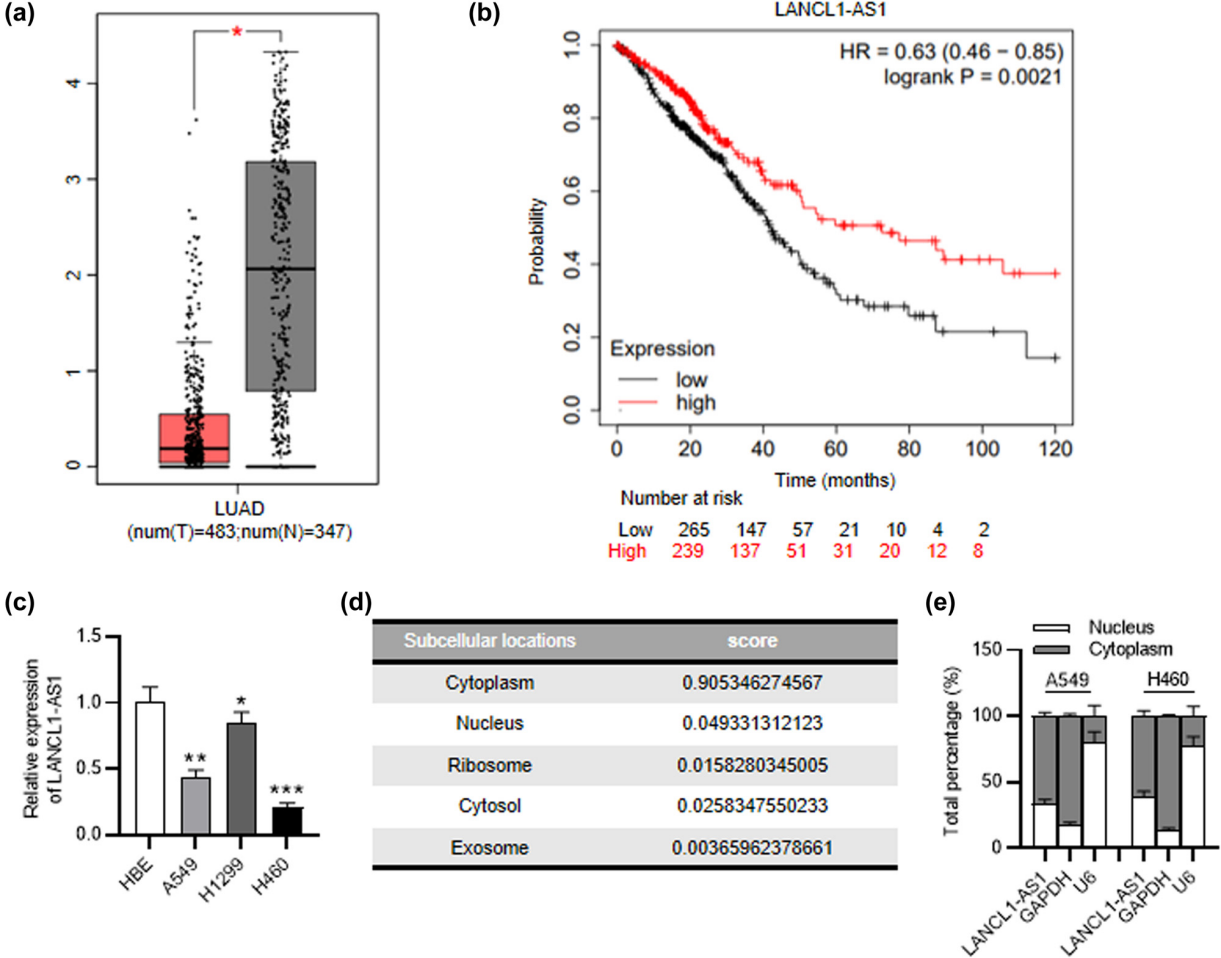
LANCL1-AS1 is downregulated in NSCLC. (a) LANCL1-AS1 expression in LUAD (*n* = 483) and normal tissues (*n* = 347) displayed by GEPIA database. (b) The relationship between LANCL1-AS1 expression and LUAD patient prognoses shown by Kaplan–Meier plotter website. (c) RT-qPCR analysis of LANCL1-AS1 level in NSCLC and normal cell lines. (d) The location of LANCL1-AS1 in cells predicted by lncLocator website. (e) Subcellular fractionation assay of LANCL1-AS1 location in NSCLC cells. ^*^ *p* < 0.05, ^**^ *p* < 0.01, ^***^ *p* < 0.001.

LANCL1-AS1 expression was examined with GEPIA (http://gepia.cancer-pku.cn/) which reveals LANCL1-AS1 downregulation in LUAD (Figure 1a). Kaplan–Meier Plotter (https://kmplot.com/analysis/) indicates that LANCL1-AS1 downregulation is associated with the poor prognosis of patients with LUAD (Figure 1b). Consistent with the above results, RT-qPCR disclosed a decreased level of LANCL1-AS1 in NSCLC cell lines (A549, H1299, and H460) in comparison to the normal cell line (HBE) (Figure 1c). In accord with the prediction of lncLocator in Figure 1d, the results of subcellular fractionation demonstrated that LANCL1-AS1 was largely located in the cytoplasm of NSCLC cells (Figure 1e). These results showed that a low level of LANCL1-AS1 is related to a poor prognosis of NSCLC.

### 3.2 Overexpression of LANCL1-AS1 restrains cell migration, invasiveness, and angiogenesis of NSCLC

**Figure 2:**
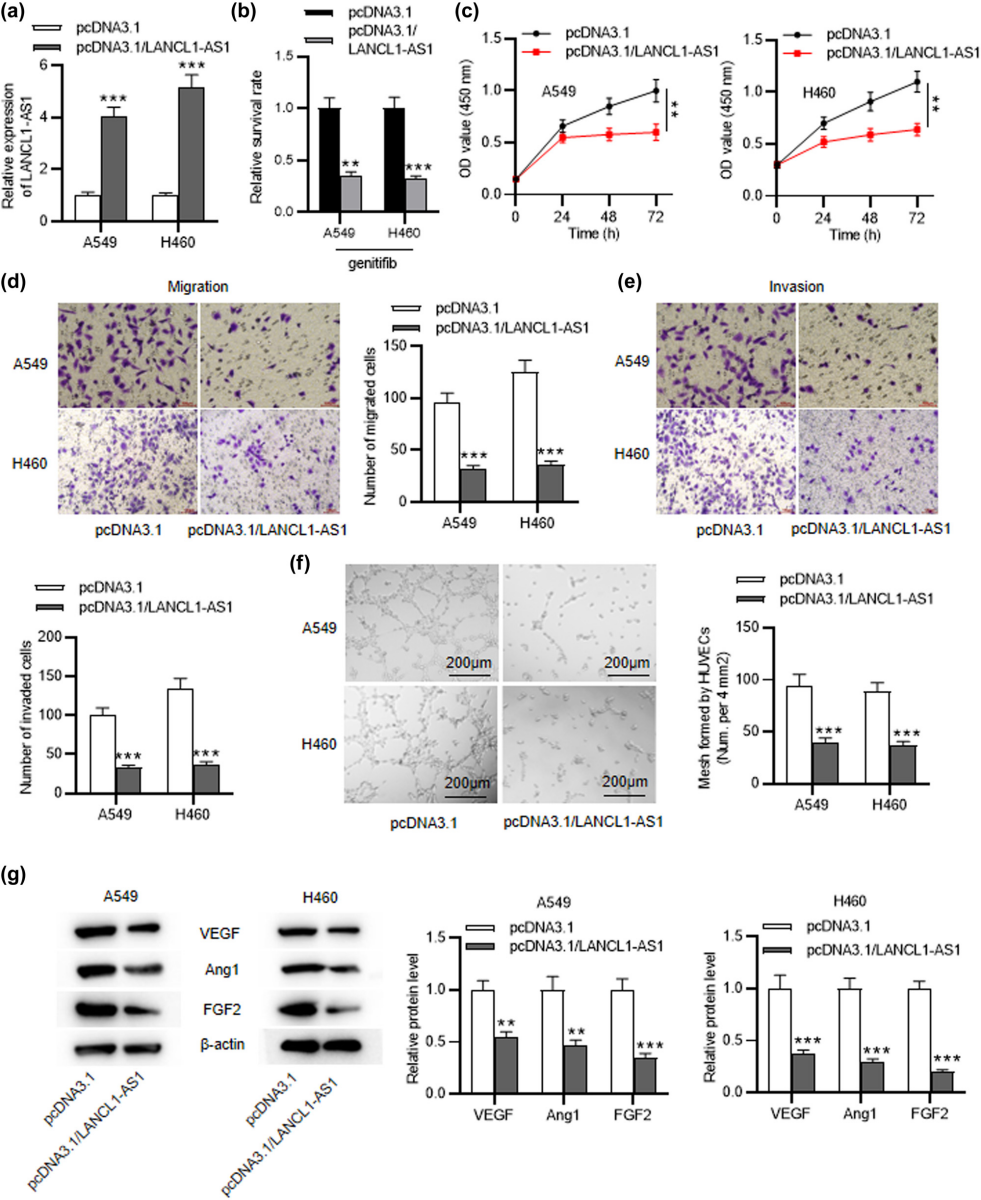
LANCL1-AS1 upregulation restrains cell migration, invasiveness, and angiogenesis of NSCLC. (a) RT-qPCR for evaluating the transfection efficiency of pcDNA3.1/LANCL1-AS1 in A549 and H460 cells. (b) The survival rate of NSCLC cells with or without LANCL1-AS1 overexpression under gefitinib treatment. (c) CCK-8 assay for evaluating the viability of LANCL1-AS1-overexpressed A549 and H460 cells. (d and e) Transwell assays for evaluating cell migratory and invasive capabilities after LANCL1-AS1 overexpression. (f) Tube formation assay for assessing angiogenic ability of HUVECs with LANCL1-AS1 upregulation. (g) Western blotting for evaluating levels of angiogenesis-related proteins. ^**^ *p* < 0.01, ^***^ *p* < 0.001.

To reveal the role of LANCL1-AS1 in NSCLC, A549 and H460 cells were stably administrated with pcDNA3.1 or pcDNA3.1/LANCL1-AS1. LANCL1-AS1 level was significantly elevated in LANCL1-AS1-overexpressed cells (Figure 2a). Then, we tested whether LANCL1-AS1 affected NSCLC cell sensitivity to the TKI drug gefitinib. Notably, under gefitinib treatment, NSCLC cells in the control group exhibited higher survival rate that those in LANCL1-AS1-overexpressed group, indicating that LANCL1-AS1 promoted NSCLC cell sensitivity to gefitinib (Figure 2b). CCK-8 assay revealed that upregulation of LANCL1-AS1 restrained NSCLC cell proliferation (Figure 2c). As displayed by Transwell assays, the migratory and invasive capabilities of NSCLC cells were suppressed after overexpressing LANCL1-AS1 (Figure 2d and e). Tube formation assay was conducted to evaluate LANCL1-AS1 impact on angiogenesis of HUVECs. The results displayed marked suppression of mesh formation using condition medium from A549 or H460 cells with LANCL1-AS1 overexpression (Figure 2f). Furthermore, expression levels of angiogenesis-related proteins (VEGF, Ang1, and FGF2) were markedly lessened in LANCL1-AS1-overexpressed A549 and H460 cells (Figure 2g). Collectively, LANCL1-AS1 restrains the migration, invasiveness, and angiogenesis of NSCLC cells.

### 3.3 LANCL1-AS1 binds to miR-3680-3p

**Figure 3:**
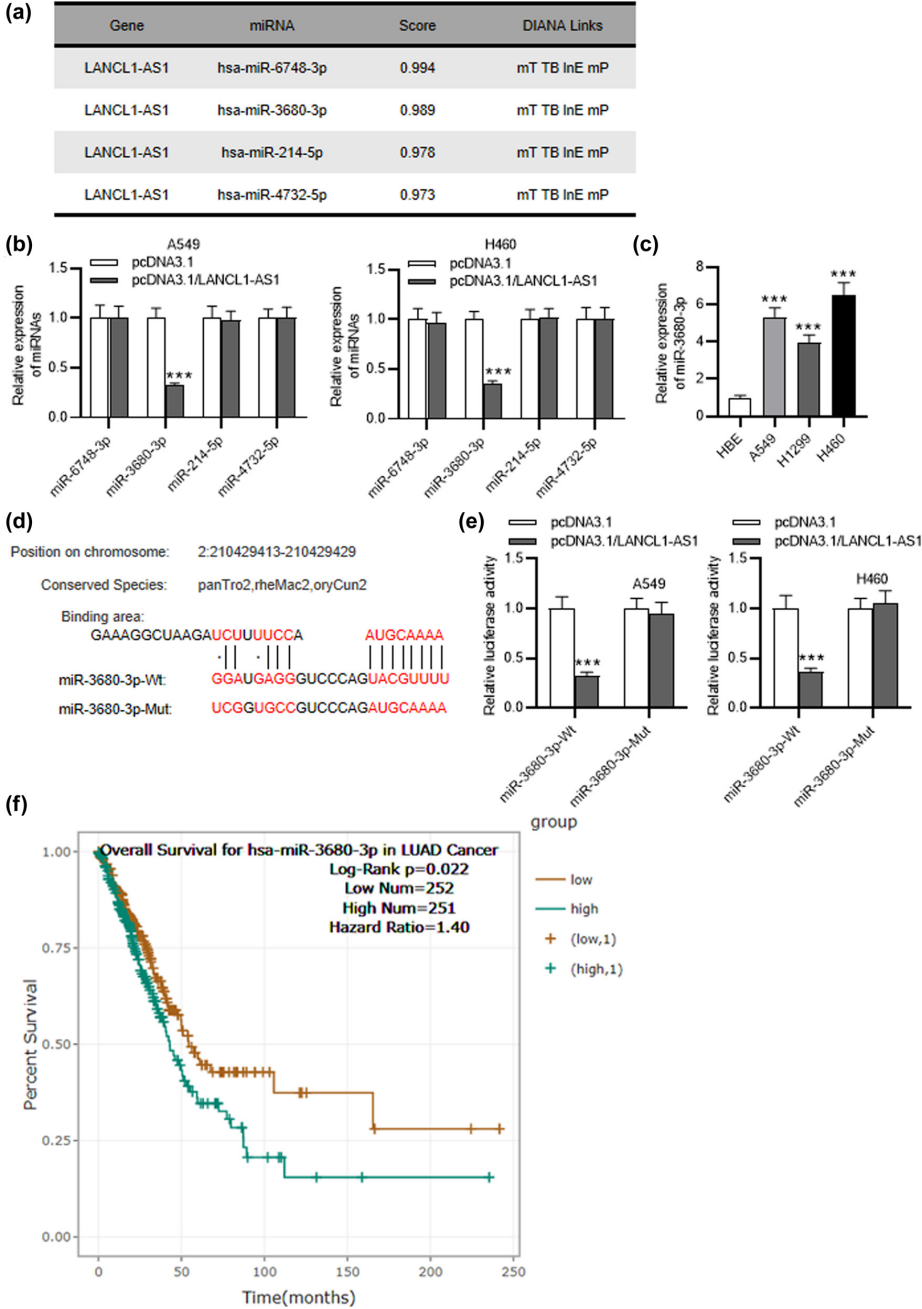
LANCL1-AS1 binds to miR-3680-3p. (a) LANCL1-AS1 downstream miRNAs predicted by DIANA. (b) RT-qPCR analysis of the miRNA levels in LANCL1-AS1-upregulated A549 and H460 cells. (c) RT-qPCR of miR-3680-3p level in NSCLC and normal cell lines. (d) Putative binding site between LANCL1-AS1 and miR-3680-3p shown by DIANA. (e) The relation between LANCL1-AS1 and miR-3680-3p verified by luciferase reporter assay. (f) The association between miR-3680-3p expression and LUAD patient prognoses shown by ENCORI. ^***^ *p* < 0.001.

DIANA website was used for screening downstream miRNAs that can interact with LANCL1-AS1. With the screening condition of score˃0.97, four miRNAs were singled out (Figure 3a). As displayed by RT-qPCR, only miR-3680-3p was significantly downregulated in LANCL1-AS1-overexpressed A549 and H460 cells (Figure 3b). Additionally, miR-3680-3p exhibited a high level in NSCLC cell lines in comparison to the normal cells (Figure 3c). DIANA predicts putative binding site between LANCL1-AS1 and miR-3680-3p (Figure 3d). Moreover, overexpression of LANCL1-AS1 was shown to decrease the luciferase activity of miR-3680-3p-Wt rather than miR-3680-3p-Mut in A549 and H460 cells (Figure 3e), verifying the binding relation between LANCL1-AS1 and miR-3680-3p. Additionally, high expression of miR-3680-3p is closely associated with the adverse prognosis of LUAD patients, as shown by ENCORI (https://starbase.sysu.edu.cn/index.php) website (Figure 3f).

### 3.4 GMFG is a target of miR-3680-3p

**Figure 4:**
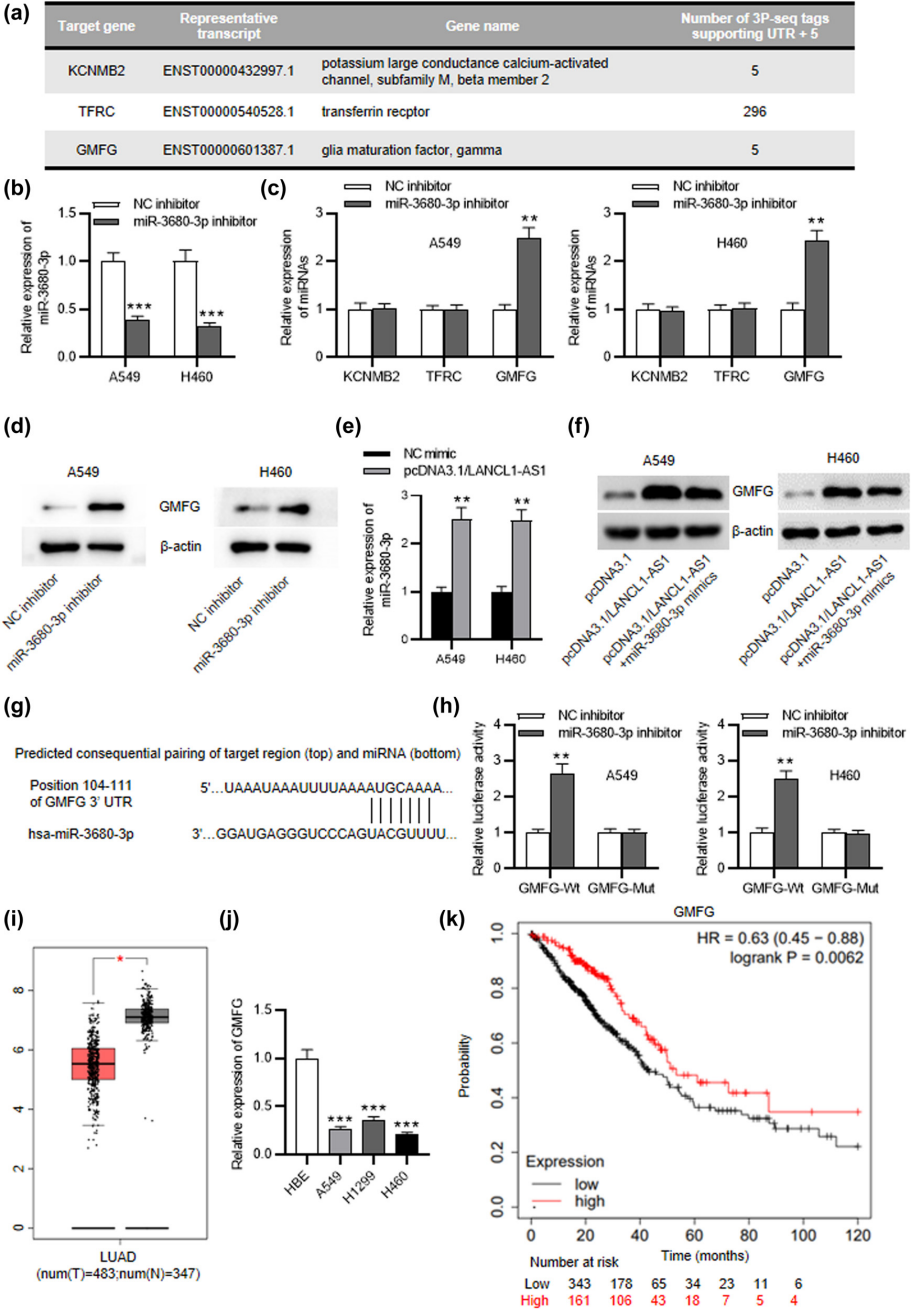
miR-3680-3p targets GMFG. (a) TargetScan predicts three candidate downstream genes of miR-3680-3p. (b) RT-qPCR for assessing miR-3680-3p inhibitor transfection efficiency. (c) RT-qPCR of mRNA expression in miR-3680-30-depleted NSCLC cells. (d) Western blotting of GMFG protein level in miR-3680-3p-depleted NSCLC cells. (e) RT-qPCR for examining miR-3680-3p overexpression efficiency. (f) Western blotting of GMFG protein level in NSCLC cells with transfection of pcDNA3.1/LANCL1-AS1 or pcDNA3.1/LANCL1-AS1 + miR-3680-3p mimics. (g) miR-3680-3p complementary site on GMFG predicted by TargetScan. (h) The binding relation between miR-3680-3p and GMFG identified by luciferase reporter assay. (i) GMFG expression in LUAD and normal tissues shown by GEPIA. (j) RT-qPCR of GMFG level in NSCLC and normal cell lines. (k) Kaplan–Meier plotter displays the association between GMFG expression and LUAD patient prognoses. ^*^ *p* < 0.05, ^**^ *p* < 0.01, ^***^ *p* < 0.001.

To further reveal the regulatory mechanism of LANCL1-AS1 in NSCLC, TargetScan was used for prediction of the downstream gene of miR-3680-3p. Three candidate mRNAs were screened out with the condition of cumulative weighted context++ score >0.7 (Figure 4a). RT-qPCR revealed the downregulation of miR-3680-3p in A549 and H460 cells treated with miR-3680-3p inhibitor (Figure 4b). Additionally, only GMFG was upregulated in miR-3680-3p-depleted A549 and H460 cells (Figure 4c). Western blotting disclosed that depletion of miR-3680-3p increased the protein level of GMFG in A549 and H460 cells (Figure 4d and e). Then, we overexpressed miR-3680-3p in NSCLC cells using miR-3680-3p mimics (Figure 4e). As shown in Figure 4f, upregulation of LANCL1-AS1 elevated GMFG protein expression in NSCLC cells, while overexpression of miR-3680-3p reversed the effect of LANCL1-AS1 upregulation. TargetScan predicts the existence of miR-3680-3p complementary site on GMFG 3'UTR and luciferase reporter assay further elucidated the binding relation between miR-3680-3p and GMFG (Figure 4g and h). GEPIA database shows the downregulation of GMFG in LUAD tissues compared with the normal samples (Figure 4i). In comparison to that in the normal cells, GMFG expression in NSCLC cells was markedly decreased (Figure 4j). Moreover, Kaplan–Meier Plotter exhibits a strong correlation between GMFG low expression and poor prognoses of LUAD patients (Figure 4k). Hence, GMFG is targeted by miR-3680-3p, and its expression is closely related to patient prognoses.

### 3.5 GMFG silencing rescues LANCL1-AS1 overexpression-mediated inhibitory effect on NSCLC cellular activities

**Figure 5:**
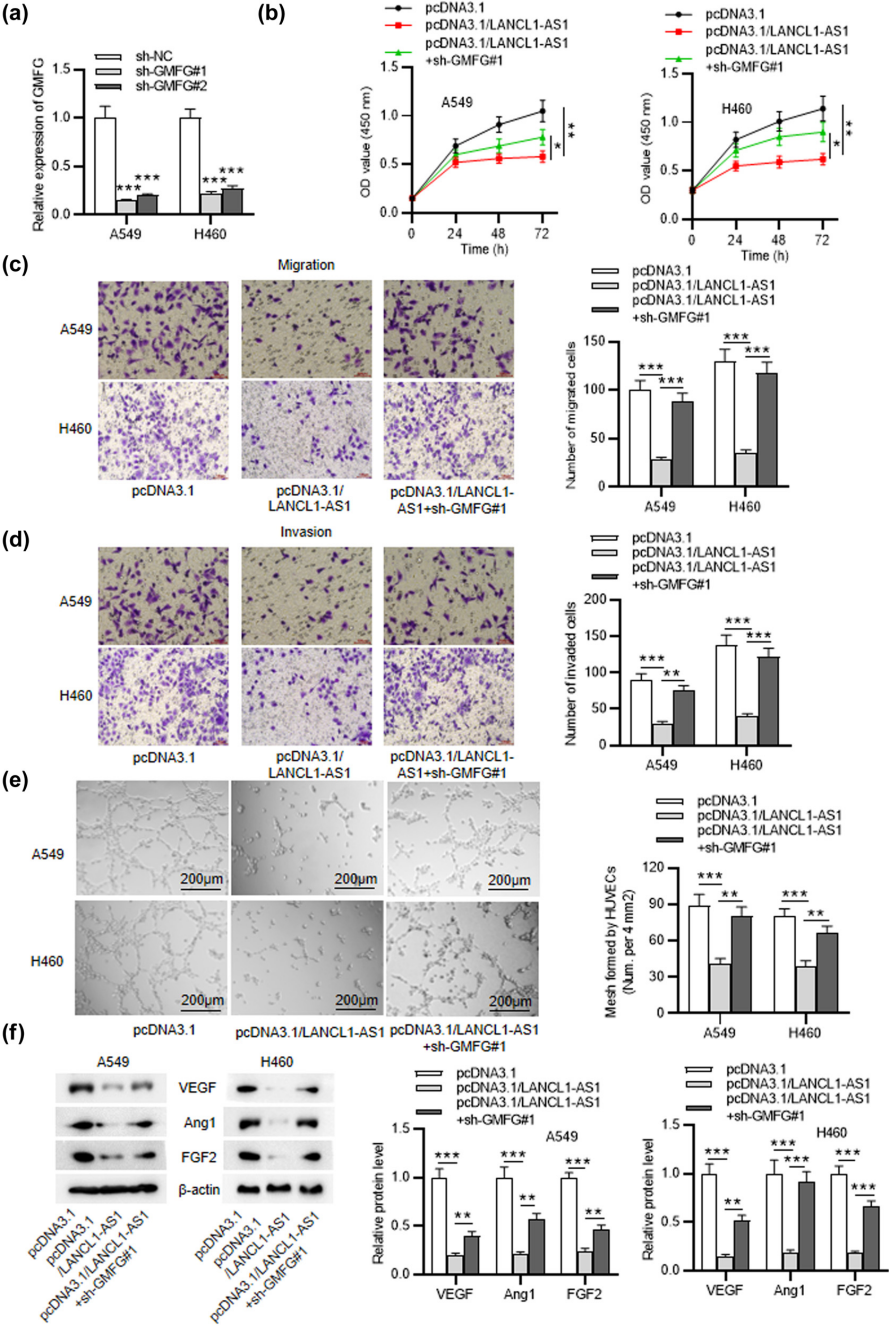
GMFG silencing rescues LANCL1-AS1 overexpression-mediated inhibitory effect on NSCLC cellular activities. (a) RT-qPCR for evaluating the transfection efficiency of sh-GMFG#1/2 in A549 and H460 cells. (b) CCK-8 assay for assessing viabilities of A549 and H460 cells treated with pcDNA3.1, pcDNA3.1/LANCL1-AS1, or pcDNA3.1/LANCL1-AS1 + sh-GMFG#1. (c and d) Transwell assays for analyzing the migration and invasiveness of NSCLC cells with above treatment. (e) Tube formation assay for detecting the mesh formed by HUVECs. (f) Western blotting for evaluating angiogenesis-related protein levels in NSCLC cells with above transfection. ^*^ *p* < 0.05, ^**^ *p* < 0.01, ^***^ *p* < 0.001.

Rescue experiments were conducted to further identify the LANCL1-AS1/miR-3680-3p/GMFG axis-mediated effect on the progression of NSCLC. The transfection efficiency of sh-GMFG#1/2 was detected by RT-qPCR (Figure 5a). As revealed by CCK-8 assay, LANCL1-AS1 overexpression-induced suppression on cell viability was partially reversed by GMFG depletion (Figure 5b). Similarly, downregulating GMFG rescued the suppression in NSCLC cell migration and invasiveness which was caused by LANCL1-AS1 upregulation (Figure 5c and d). Moreover, tube formation assay and western blotting displayed that LANCL1-AS1 overexpression-induced inhibitory impact on angiogenesis of HUVECs was significantly attenuated by GMFG knockdown (Figure 5e and f). Collectively, LANCL1-AS1 inhibits NSCLC progression via the miR-3680-3p/GMFG axis.

### 3.6 Overexpression of LANCL1-AS1 inhibits tumorigenesis of NSCLC *in vivo*

**Figure 6:**
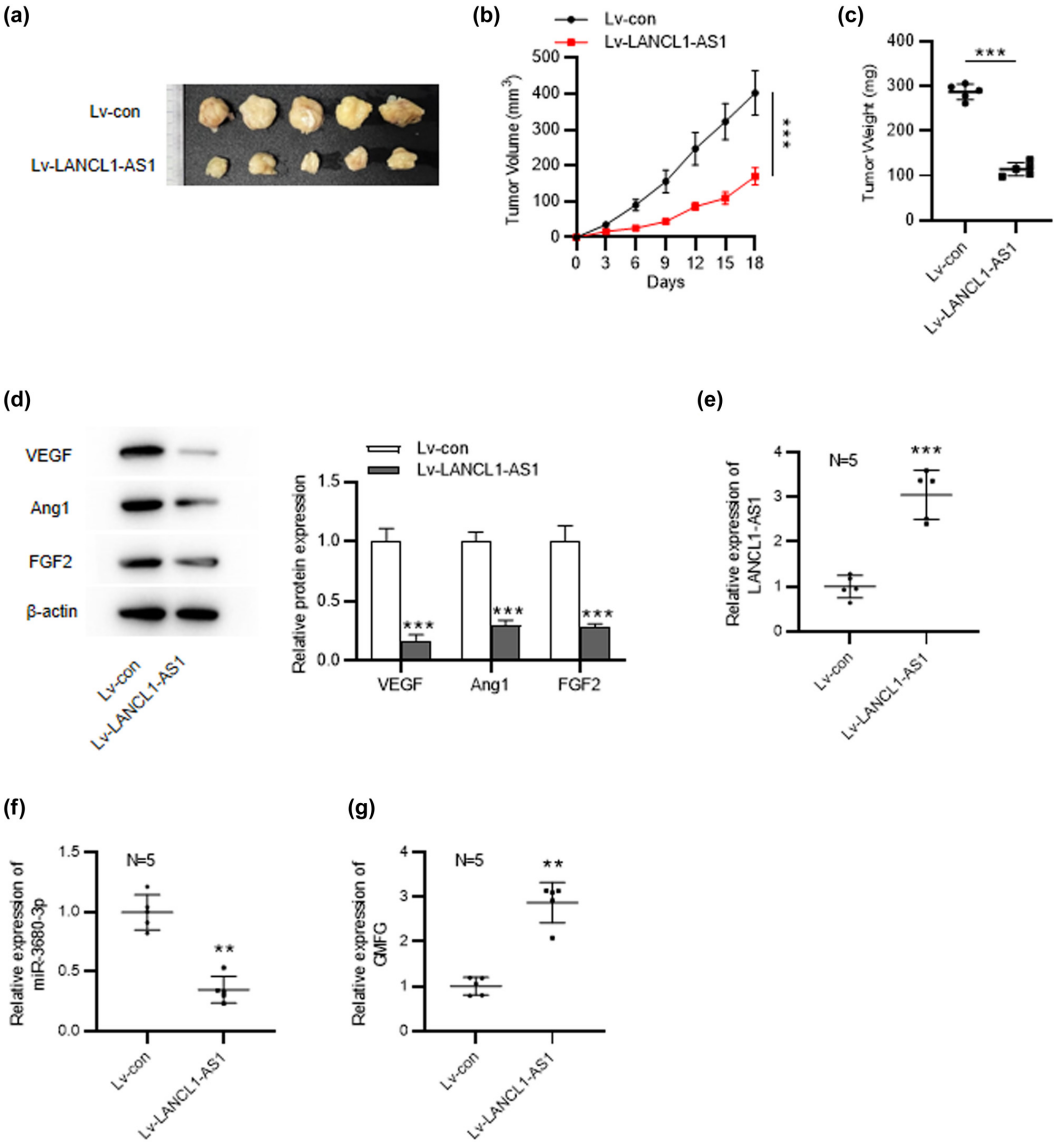
LANCL1-AS1 overexpression inhibits tumorigenesis of NSCLC *in vivo*. (a) BALB/c nude mice were implanted with H460 cells stably expressing Lv-pcDNA3.1 or Lv-LANCL1-AS1. *n* = 5 per group. (b) Tumor volumes were detected every 3 days. (c) Tumor weight was measured on the 18th day. (d) Western blotting for examining levels of angiogenesis-related proteins in tumors. (e–g) RT-qPCR analysis for evaluating LANCL1-AS1, miR-3680-3p, and GMFG expression in tumor tissues. ^**^ *p* < 0.01, ^***^ *p* < 0.001.

To further elucidate LANCL1-AS1 effect on NSCLC, *in vivo* experiments were carried out. Results displayed that the tumors in LANC1-AS1-overexpressed group grew much more slowly and smaller than those in the control group (Figure 6a–c). Notably, overexpression of LANCL1-AS1 markedly inhibited the expression of angiogenesis-associated proteins in tumors (Figure 6d). These indicated that LANCL1-AS1 exerts an inhibitory effect on tumorigenesis of NSCLC. Additionally, we detected the expression levels of LANCL1-AS1, miR-3680-3p, and GMFG in the tumors of each group. As depicted by the results, LANCL1-AS1 displayed a significantly higher level in Lv-LANCL1-AS1-treated group than that in the control group, confirming the successful overexpression of LNACL1-AS1 in the tumor xenograft mouse model (Figure 6e). Moreover, miR-3680-3p was downregulated and GMFG was markedly upregulated in LANCL1-AS1-overexpressed group (Figure 6f and g). These data indicated that LANCL1-AS1 upregulated GMFG expression by interacting with miR-3680-3p *in vivo*.

## 4 Discussion

In the present study, we found that lncRNA LANCL1-AS1 acted as a tumor suppressor in NSCLC by regulating the miR-3680-3p/GMFG axis. Our data showed that overexpression of LANCL1-AS1 inhibited NSCLC cell proliferation, migration, invasion as well as angiogenesis. This suppressive effect on the tumorigenesis of NSCLC was also verified in tumor-bearing mouse models.

Despite the progress made in the diagnoses and treatments, the incidence of NSCLC is still high, with the overall 5-year survival rate lower than 17% [12]. Emerging evidence has illustrated the prominent effects of lncRNAs involved in regulation of cancers, including NSCLC. Multiple lncRNAs are considered as a prognostic biomarker and therapeutic target in NSCLC. For example, RP11-909N17.2 facilitates the cellular processes of NSCLC and predicts an adverse prognosis of patients [13]. PCAT29 inhibits NSCLC progression via the miR-494/PTEN axis and NSCLC patients with a higher level of PCAT29 have a better prognosis [14]. These suggest that lncRNAs can be a tumor suppressor gene or an oncogene. Here we probed the function of LANCL1-AS1 in NSCLC. LANCL1-AS1 is a novel gene that has been indicated to be downregulated in NSCLC; however, to our knowledge, the detailed functions of LANCL1-AS1 have not been studied yet. Currently, LANCL1-AS1 was shown to be downregulated in NSCLC cell lines and bioinformatics analysis revealed that its downregulation is associated with the adverse survival of LUAD patients, indicating the tumor suppressor role of LANCL1-AS1 in NSCLC. Moreover, gain-of-function assays demonstrated that LANCL1-AS1 overexpression restrained the proliferation, motion, and angiogenesis of NSCLC cells *in vitro*. Importantly, *in vivo* experiments displayed that upregulated LANC1-AS1 restrained tumorigenesis and angiogenesis in mice. These results elucidated that LANCL1-AS1 suppresses NSCLC progression.

Numerous studies have elucidated that in the cytoplasm, lncRNAs function as a ceRNA to competitively absorb microRNAs (miRNAs) and subsequently regulate messenger RNA (mRNA) expression [15]. For example, in prostate cancer, LINC01679 suppresses tumor progression by regulating miR-3150a-3p/SLC17A9 axis [16]. Additionally, in thyroid cancer, MIAT contributes to tumor progression by absorbing miR-150-5p and modulate EZH2 [17]. Bioinformatics analysis and our assays confirmed that LANCL1-AS1 was largely distributed in the cytoplasm of NSCLC cells, indicating the potential of LANCL1-AS1 as a ceRNA in NSCLC. With the use of bioinformatic tools and a series of experiments, we finally identified miR-3680-3p that could bind with LANCL1-AS1 in NSCLC cells. miR-3680-3p was shown to be sponged by circ-PRKCI to regulate AKT3 expression in esophageal squamous cell carcinoma [18]. Presently, it was shown that miR-3680-3p was upregulated in NSCLC cells and its upregulation correlates with the adverse survival of LUAD patients.

It is recognized that miRNAs can bind to mRNA 3'UTRs by base-pairing, consequently leading to either mRNA degradation or translation suppression [19]. To better understand the mechanism of LANCL1-AS1, we screened the downstream gene of miR-3680-3p. Eventually, GMFG was singled out which displayed downregulated expression in NSCLC and strongly associated with the prognosis of LUAD patients. GMFG has been indicated to play a part in several cancers. For example, a high level of GMFG is related to adverse prognosis and facilitates the progression of epithelial ovarian cancer [20]. Moreover, GMFG exerts an antitumor role in breast cancer and is considered as a promising biomarker for the diagnosis and prognosis [21]. Intriguingly, GMFG was indicated to be downregulated in LUAD [22]. In this study, GMFG silencing was shown to reverse LANCL-AS1 overexpression-mediated inhibitory impact on NSCLC cell malignant behaviors, indicating the antitumor effect of the LANCL1-AS1/miR-3680-3p/GMFG axis in NSCLC.

In conclusion, we probed the role of LANCL1-AS1 in NSCLC. LANCL1-AS1 suppresses the migration, invasiveness, and angiogenesis of NSCLC cells and inhibits tumorigenesis *in vivo*. Mechanistically, LANCL1-AS1 exerts its antitumor effect by functioning as a ceRNA via the miR-3680-3p/GMFG axis. The findings might develop a novel idea for the therapy of NSCLC.

## References

[1] Zhao Z, Wan J, Guo M, Yang Z, Li Z, Wang Y, et al. Long non-coding RNA LINC01559 exerts oncogenic role via enhancing autophagy in lung adenocarcinoma. Cancer Cell Int. 2021;21(1):624. 10.1186/s12935-021-02338-4 PMC8614059 34823534

[2] Siegel RL, Miller KD, Fuchs HE, Jemal A. Cancer statistics, 2022. CA Cancer J Clin. 2022;72(1):7–33. 10.3322/caac.21708 35020204

[3] Cruz CRV, Ferrer JLM, Garcia RL. Concomitant and decoupled effects of cigarette smoke and SCAL1 upregulation on oncogenic phenotypes and ROS detoxification in lung adenocarcinoma cells. Sci Rep. 2021;11(1):18345. 10.1038/s41598-021-97869-1 PMC8443756 34526564

[4] Liu S, Zhan N, Gao C, Xu P, Wang H, Wang S, et al. Long noncoding RNA CBR3-AS1 mediates tumorigenesis and radiosensitivity of non-small cell lung cancer through redox and DNA repair by CBR3-AS1/miR-409-3p/SOD1 axis. Cancer Lett. 2021;526:1–11. 10.1016/j.canlet.2021.11.009 34801596

[5] Siegel RL, Miller KD, Fuchs HE, Jemal A. Cancer Statistics, 2021. CA Cancer J Clin. 2021;71(1):7–33. 10.3322/caac.21654 33433946

[6] Bútová R, Vychytilová-Faltejsková P, Gregorová J, Radová L, Almáši M, Bezděková R, et al. LncRNAs LY86-AS1 and VIM-AS1 distinguish plasma cell leukemia patients from multiple myeloma patients. Biomedicines. 2021;9(11):1637. 10.3390/biomedicines9111637 PMC8615960 34829867

[7] Chen X, Song J, Wang X, Sun D, Liu Y, Jiang Y. LncRNA LINC00460: Function and mechanism in human cancer. Thorac cancer. 2021;13(1):3–14. 10.1111/1759-7714.14238 PMC8720622 34821482

[8] Zhu B, Finch.-Edmondson M, Leong KW, Zhang X, V M, Lin QXX, et al. LncRNA SFTA1P mediates positive feedback regulation of the Hippo-YAP/TAZ signaling pathway in non-small cell lung cancer. Cell Death Discovery. 2021;7(1):369. 10.1038/s41420-021-00761-0 PMC8630011 34845189

[9] Li Y, Zhang H, Guo J, Li W, Wang X, Zhang C, et al. Downregulation of LINC01296 suppresses non-small-cell lung cancer via targeting miR-143-3p/ATG2B. Acta Biochim Biophys Sin. 2021;53(12):1681–90. 10.1093/abbs/gmab149 34695177

[10] Bai Y, Long J, Liu Z, Lin J, Huang H, Wang D, et al. Comprehensive analysis of a ceRNA network reveals potential prognostic cytoplasmic lncRNAs involved in HCC progression. J Cell Physiol. 2019;234(10):18837–48. 10.1002/jcp.28522 PMC6618076 30916406

[11] Acha-Sagredo A, Uko B, Pantazi P, Bediaga NG, Moschandrea C, Rainbow L, et al. Long non-coding RNA dysregulation is a frequent event in non-small cell lung carcinoma pathogenesis. Br J Cancer. 2020;122(7):1050–8. 10.1038/s41416-020-0742-9 PMC7109049 32020063

[12] He Y, Jiang X, Duan L, Xiong Q, Yuan Y, Liu P, et al. LncRNA PKMYT1AR promotes cancer stem cell maintenance in non-small cell lung cancer via activating Wnt signaling pathway. Mol Cancer. 2021;20(1):156. 10.1186/s12943-021-01469-6 PMC8638142 34856993

[13] Tuo Z, Zhang A, Ma L, Zhou Z. Long noncoding RNA RP11-909N17.2 presages a poor prognosis of non-small cell lung cancer. Cancer Biomark Sect A Dis Markers. 2022;34(2):211–9. 10.3233/CBM-203263 PMC12364280 34957995

[14] Lu B, Lv H, Yang Z, Shu J, Zhang H. LncRNA PCAT29 up-regulates the expression of PTEN by down-regulating miR-494 in non-small-cell lung cancer to suppress tumor progression. Crit Rev Eukaryot Gene Expr. 2021;31(6):9–15. 10.1615/CritRevEukaryotGeneExpr.2021039081 34936288

[15] Fernandes M, Marques H, Teixeira AL, Medeiros R. Competitive endogenous RNA network involving miRNA and lncRNA in non-hodgkin lymphoma: Current advances and clinical perspectives. Biomedicines. 2021;9(12):1934. 10.3390/biomedicines9121934 PMC8698845 34944752

[16] Mi YY, Sun CY, Zhang LF, Wang J, Shao HB, Qin F, et al. Long non-coding RNAs LINC01679 as a competitive endogenous RNAs inhibits the development and progression of prostate cancer via regulating the miR-3150a-3p/SLC17A9 axis. Front Cell Dev Biol. 2021;9:737812. 10.3389/fcell.2021.737812 PMC8656699 34900992

[17] Guo K, Qian K, Shi Y, Sun T, Wang Z. LncRNA-MIAT promotes thyroid cancer progression and function as ceRNA to target EZH2 by sponging miR-150-5p. Cell Death Dis. 2021;12(12):1097. 10.1038/s41419-021-04386-0 PMC8608816 34811354

[18] Shi N, Shan B, Gu B, Song Y, Chu H, Qian L. Circular RNA circ-PRKCI functions as a competitive endogenous RNA to regulate AKT3 expression by sponging miR-3680-3p in esophageal squamous cell carcinoma. J Cell Biochem. 2019;120(6):10021–30. 10.1002/jcb.28285 30659640

[19] Marima R, Francies FZ, Hull R, Molefi T, Oyomno M, Khanyile R, et al. MicroRNA and alternative mRNA splicing events in cancer drug response/resistance: Potent therapeutic targets. Biomedicines. 2021;9(12):1818. 10.3390/biomedicines9121818 PMC8698559 34944633

[20] Zuo P, Ma Y, Huang Y, Ye F, Wang P, Wang X, et al. High GMFG expression correlates with poor prognosis and promotes cell migration and invasion in epithelial ovarian cancer. Gynecol Oncol. 2014;132(3):745–51. 10.1016/j.ygyno.2014.01.044 24486602

[21] Yang Y, He X, Tang QQ, Shao YC, Song WJ, Gong PJ, et al. GMFG has potential to be a novel prognostic marker and related to immune infiltrates in breast cancer. Front Oncol. 2021;11:629633. 10.3389/fonc.2021.629633 PMC8343142 34367945

[22] Lan A, Ren C, Wang X, Tong G, Yang G. Bioinformatics and survival analysis of glia maturation factor-γ in pan-cancers. BMC Cancer. 2021;21(1):423. 10.1186/s12885-021-08163-2 PMC8052856 33863293

